# Clinical outcomes of a beveled tip, ultra-high speed, 25-gauge pars plana vitrectomy system

**DOI:** 10.1186/s12886-022-02311-3

**Published:** 2022-02-24

**Authors:** Harvey S. Uy, Vicente Lorenzo O. Cabahug, Jose Carlo M. Artiaga, Pik Sha Chan, Jordan T. Famadico

**Affiliations:** 1 Peregrine Eye and Laser Institute, Morning Star Center, 347 Gil Puyat Avenue, Bel Air, 1209 Makati City, Philippines; 2 Department of Ophthalmology and Visual Sciences, University of the Philippines, Manila, Philippines; 3 Eye Institute, St Luke’s Medical Center, Quezon City, Philippines; 4 Cebu Doctors’ University Hospital, Cebu City, Philippines; 5 Moorfields Eye Hospital NHS Foundation Trust, London, UK; 6 Mary Mediatrix Medical Center, Lipa City, Philippines

**Keywords:** Pars plana vitrectomy, Microincisional vitrectomy surgery, MIVS, Ultra-high speed vitrectomy, Beveled tip cutter probe

## Abstract

**Objective:**

To report the clinical outcomes of a 25-gauge, beveled-tip, 10,000 cuts-per-minute (cpm) microincisional vitrectomy surgery (MIVS) system.

**Methods:**

Prospective case series of eyes undergoing primary pars plana vitrectomy (PPV) for common vitreoretinal indications. Main outcome measures were: rate of achieving surgical objectives, operative times, number of surgical steps, use of ancillary instruments, corrected distance visual acuity (CDVA), and adverse events (AE).

**Results:**

The surgical objectives were achieved in all eyes. Mean total operative time (TOT), core, shave and total vitrectomy times were 1891 ± 890, 204 ± 120, 330 ± 320, 534 ± 389 s, respectively. Mean number of surgical steps was 4.3 ± 1.5. Mean number of ancillary instruments used was 4.5 ± 1.9. Mean CDVA improved by 0.53 ± 0.56 logMAR units (*P* < 0.001) 3 months postoperatively. AE included elevated IOP (8%), hypotony (6%), and re-detachment (2%). Majority (82%) had no postoperative discomfort. The number of surgical steps demonstrated a positive correlation with TOT (*p* < 0.05), number of ancillary instruments used (*p* < 0.05), and postoperative Day 1 IOP (*p* < 0.05). The number of times ancillary instrumentation was used demonstrated a positive correlation with TOT (*p* < 0.05).

**Conclusion:**

Beveled-tip, 10,000 cpm MIVS system effectively and safely performs common VR procedures of varying complexity and may reduce operative times and use of ancillary instrumentation.

**Supplementary Information:**

The online version contains supplementary material available at 10.1186/s12886-022-02311-3.

## Introduction

In the 1970’s, Robert Machemer performed the first closed-system, pars plana vitrectomy (PPV) using a single-port, 17-gauge (17G) system with a maximal cut rate of 400 cuts per minute (cpm) [1]. Since then, the efficacy, efficiency and safety of PPV has improved with the introduction of microincisional vitrectomy surgery (MIVS), wide-angle viewing as well as new surgical techniques such as membrane dissection, internal subretinal fluid drainage, fluid-air exchange (FAX), and endophotocoagulation [2–5].

**Fig. 1:**
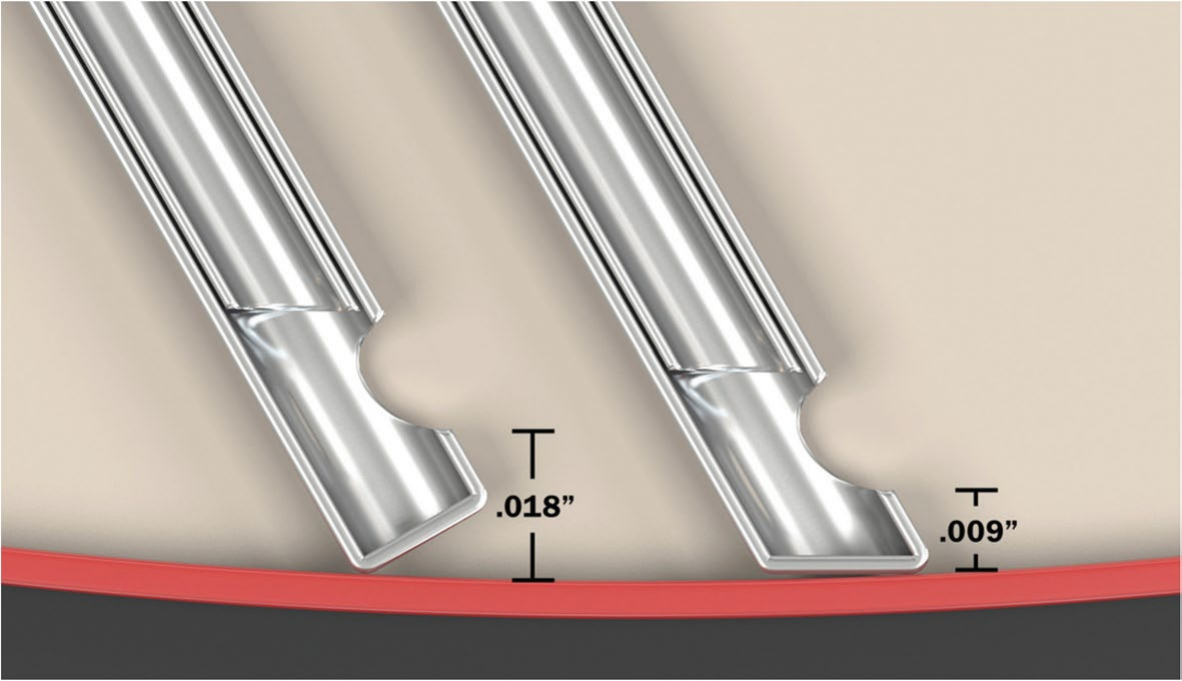
Schematic of the Beveled probe (right) compared with the conventional probe design *(Alcon Laboratories, Fort Worth, TX, USA)*

Smaller gauge probes, faster cut rates and fluidics control have increased PPV precision, decreased operative times, hastened postoperative recovery and reduced postoperative pain and complications [6, 7]. A recently introduced beveled-tip cutter probe (BTCP) with shortened port-tip distance potentially facilitates access to surgical tissue planes, permits an expanded range of surgical maneuvers and performs multiple functions (Fig. 1) [4, 5]. There are few publications describing the clinical use of this relatively novel cutter probe configuration; fewer still are publications that report on the multifunctional capabilities of cutter probes and how they might potentially enhance efficiency by reducing the usage of ancillary instrumentation. The purpose of this study was to describe the clinical outcomes and assess the utility of using a 25-gauge (25G), 10,000 cpm BTCP for the treatment of various vitreoretinal (VR) diseases.

## Patients and methods

This is a single-center, prospective case series of 50 eyes that underwent primary PPV performed at the Peregrine Eye and Laser Institute (PELI) from April 24 to October 24, 2019. The study was conducted according to the tenets of the Declaration of Helsinki. The study protocol and informed consent form was approved by the PELI-Institutional Review Board. Participants provided informed consent prior to enrollment. We included eyes undergoing primary PPV for non-resolving vitreous hemorrhage (VH) and vitritis, clinically significant epiretinal membranes (ERM), clinically significant lamellar and full thickness macular holes (MH), vitreo-macular traction (VMT), rhegmatogenous retinal detachment (RRD) or tractional retinal detachment (TRD), retained lens fragments (RLF), and dislocated intraocular lenses (DIS). We excluded eyes with history of glaucoma, prior glaucoma surgery, scleral thinning, recent (< 3 months) ocular infection, central corneal opacification which would affect surgical visualization, clinically significant crystalline lens opacities where the patient did not agree to simultaneous lens removal, non-dilating pupils (< 5 mm with maximal dilation) and choroidal detachment.

The patients underwent comprehensive ophthalmologic assessment including corrected distance visual acuity (CDVA), intraocular pressure (IOP) measurement, slit-lamp and dilated fundus examination at all visits. IOP was measured using applanation tonometry during clinic visits and digital contact tonometry during surgery (Tonopen AVIA, Reichert Technologies, Depew, NY, USA). Elevated IOP was defined as > 22 mmHg while hypotony was defined as < 5 mmHg.

PPV was performed using 25G, 10,000 cpm BTCP (25G Advanced Ultravit, CONSTELLATION Vision System, Alcon Surgical, Irvine, CA, USA) by a trained retinal surgeon (HSU, PCU or JTF). A wide-angle viewing system (Resight 700, Zeiss Medical Technology, Jena, Germany) and high magnification contact lens (HR Direct High Mag Surgical Lens, Volk Optical, Inc., Mentor, OH, USA) provided surgical visualization. Trocars were inserted 3.5 mm from the limbus. Central vitreous was removed using the core vitrectomy setting (open-biased, IOP = 25 mmHg, maximum vacuum = 650 mmHg, cut-rate = 10,000 cpm); peripheral vitreous was removed using shave vitrectomy setting (closed-biased, IOP = 25 mmHg, maximum vacuum = 300 mmHg, cut rate = 10,000 cpm).

For membrane peeling, visualization was enhanced using Brilliant Blue, Trypan Blue and soluble lutein (Doubledyne, Horus Pharma, St. Laurent du Var, France) which was refluxed onto the retinal surface using the BTCP. Initial ERM or internal limiting membrane (ILM) flap edges were created using ILM forceps or nitinol loops. Whenever safely possible, the BTCP was used to grasp the flap edge to perform membrane removal, otherwise forceps was used to complete the process.

For RRD, the 25G BTCP was used to aspirate subretinal fluid through extramacular pre-existing breaks or drainage retinotomies. Small residual amounts of fluid on the macular or optic nerve head surface were aspirated using a soft-tip, backflush handpiece (25-gauge, Grieshaber Advanced Backflush DSP, Alcon Surgical, Fort Worth, TX, USA). Endophotocoagulation was applied around the retinal breaks and in cerclage fashion along the equatorial region followed by placement of tamponade agents. The patients were typically followed up on postoperative Day 1, Week 1, Month 1 and Month 3.

The main outcome measures were rate of achievement of surgical objectives, total operative time (TOT) from first trocar insertion to last trocar removal, core vitrectomy time (CVT), shave vitrectomy time (SVT), total vitrectomy time (TVT = CVT + SVT) as recorded by the circulating nurse using a stopwatch, perioperative IOP, number of surgical steps, number of times ancillary instrumentation was placed into the eye, CDVA, and adverse events (AE). The number of surgical steps referred to the number of maneuvers that were executed: PPV, ERM or ILM peeling, FAX, endolaser treatment, gas or silicon oil tamponade, amniotic membrane application, lens material or IOL removal, and secondary IOL implantation. Pain at Postoperative Day 1 was reported by the patient using the following ordinal scale: 0 - no pain; 1 - mild pain not requiring medication; 2 - moderate pain less than half of waking day requiring medication; 3 - moderate pain more than half of waking day requiring medication; 4 - pain that is interrupting sleep and requiring medication.

Descriptive analysis was used for continuous and interval variables. Correlation analysis was also applied, where Pearson *r* coefficient was applied for interval level of data, while Spearman *r* rank coefficient for ordinal level of data such as pain scores. Level of significance is at 5%. Medcalc Statistical software version 19.2.1 was utilized for statistical calculations.

## Results

Fifty consecutive eyes underwent PPV for various indications (Table 1). The mean patient age was 57.2 ± 13.5 (range, 16–84). Baseline CDVA ranged from 20/20 to light perception vision with a mean CDVA of 1.24 ± 0.88 logMAR units. The mean preoperative IOP was 13.2 ± 4.2 (range, 4–30) mmHg. The surgical indications were MH (22%), RRD (20%), vitreous hemorrhage (20%), ERM (12%), TRD (10%), DIS (6%), vitritis (6%) and RLF (4%). All RRD eyes were macula-off and all TRD eyes had foveal involvement as confirmed by optical coherence tomography. Thirteen (26%) had multiple surgical indications: MH + ERM (8%), VH + ERM (6%), TRD + VH (4%), TRD + Vitritis (2%), RRD + VH (2%), dislocated IOL + VH (2%) and VH + retinal tear (2%).

**Table 1:** Beveled tip, ultra-high speed, 25-gauge pars plana vitrectomy system study data

Patient	Age (yrs)	Gender	Diagnosis	Surgical steps	# Steps	Core time (secs)	Shave time (secs)	PPV time (secs)	Total time (secs)
1	29	M	VH	PPV, LX	2	186	180	366	756
2	69	F	MH + ERM	PPV, ERM, ILM, LX, FAX, GT	6	372	177	549	2130
3	56	M	RRD	PPV, FAX, LX, GT	4	474	612	1086	2526
4	62	M	RRD	PPV, FAX, LX, GT	4	300	708	1008	1956
5	61	F	VH	PPV, LX	2	240	360	600	1350
6	57	F	RRD	PPV, FAX, LX, GT	4	120	978	1098	2676
7	64	M	MH + ERM	PPV, ERM, ILM, FAX, LX, GT	6	270	549	819	3636
8	34	M	RRD	PPV, FAX, LX, ST	4	246	882	1128	3078
9	38	M	VH + ERM	PPV, ERM, FAX, LX	4	234	296	530	1506
10	69	M	MH	PPV, ERM, ILM, FAX, LX, GT, AM	7	474	416	890	3786
11	55	F	VH + ERM	PE, PPV, ERM, LX, FAX	5	246	302	548	1920
12	74	F	DIS + VH	PPV, LX, RIOL	3	120	120	240	1686
13	53	F	TRD + VH	PE, PPV, ERM, LX, FAX, LX	6	594	1782	2376	3726
14	52	M	TRD + VH	PE, PPV, ERM	3	474	119	593	2250
15	59	M	RRD + VH	PPV, ERM, FAX, LX	4	180	183	363	810
16	54	M	MH, ERM	PPV, ERM, FAX, LX	4	120	180	300	1890
17	16	F	RRD	PPV, ERM, FAX, LX, GT	5	180	400	580	3930
18	63	M	VH + ERM	PPV, ERM, FAX, LX	4	120	120	240	1230
19	77	F	ERM	PPV, ERM, LX, FAX	4	180	312	492	1398
20	63	M	VH	PPV, LX	2	297	408	705	1140
21	84	F	VIT	PPV, LX	2	238	246	484	798
22	42	M	DIS	PPV, LX, RIOL, SIOL	4	241	180	421	2118
23	70	M	VH + RT	PPV, LX, FAX	3	180	234	414	768
24	61	F	ERM	PPV, ERM, LX, FAX	5	121	120	241	1446
25	69	F	ERM	PPV, ERM, LX, FAX	4	119	121	239	1026
26	74	M	RLF	PPV	1	293	120	413	510
27	60	M	RRD	PPV, FAX, LX, GT	4	120	1188	1308	2640
28	47	F	VH, TRD	PPV, ERM, ILM, FAX, LX, GT	6	236	426	662	3420
29	84	F	MH, ERM	PPV, ERM, ILM, FAX, LX, GT	6	60	240	300	2130
30	56	M	RRD	PPV, PE, PCIOL, ERM, FAX, LX, GT	7	359	546	905	2682
31	34	M	TRD + VIT	PPV, PE, PCIOL, LX	4	240	300	540	708
32	63	M	RRD	PPV, FAX, LX, GT	4	240	492	732	1668
33	64	F	MH	PPV, ERM, LX, FAX	4	120	240	360	2676
34	52	F	VIT	PPV, MD, FAX	3	301	240	541	1686
35	56	M	ERM	PPV, ERM, LX, FAX	4	120	120	240	1728
36	59	M	DIS	PPV, LX, RIOL	3	90	90	180	2010
37	62	F	MH	PPV, PE, PCIOL, ERM, ILM, FAX, LX, AM, GT	9	105	360	465	2286
38	38	F	VH	PPV, MD, FAX	3	138	144	282	768
39	51	F	MH	PPV, ERM, ILM, FAX, LX	5	202	120	322	1116
40	66	F	MH	PPV, ERM, ILM, FAX, LX, GT	6	150	180	330	2076
41	62	F	RRD	PPV, FAX, LX, GT	4	83	589	672	2190
42	64	M	ERM	PPV, ERM, ILM, FAX	4	120	120	240	1308
43	65	M	MH	PPV, ERM, ILM, FAX, LX	5	72	165	237	1866
44	44	M	VH	PPV, ERM, LX, FAX	4	90	120	210	918
45	63	F	ERM	PPV, ERM, ILM, FAX, LX	5	120	120	240	1140
46	38	M	RLF	PPV, LX	2	60	95	155	1425
47	55	F	TRD	PPV, PE, PCIOL, ERM, LX, FAX	6	180	150	330	1758
48	62	F	MH	PPV, ERM, ILM, FAX, LX	5	120	120	240	2250
49	52	M	VH	PPV, LX, FAX	3	60	120	180	930
50	56	M	TRD + VH	PPV, PE, PCIOL, LX, ERM, FAX	6	180	120	300	3120
**Mean**	***57.2***				***4.3***	***204***	***330***	***534***	***1891***
**SD**	***13.5***				***1.5***	***120***	***320***	***389***	***890***

Patient	IOP Day 1	# Times ancillary Instrument placed in eye	Objective achieved?	Wound leak?	Pain score (0–4)	Preoperative DCVA	Month 3 DCVA	logMAR Change	Adverse Events	Comments
1	4	2	Y	N	0	1.854	**0.398**	**−1.456**	N	
2	8	8	Y	N	0	2.301	**1.000**	**−1.301**	N	
3	18	4	Y	N	0	2.602	**2.301**	**−0.301**	N	
4	10	3	Y	N	0	2.602	**1.301**	**−1.301**	N	
5	8	2	Y	N	0	1.854	**0.398**	**−1.456**	N	
6	18	3	Y	N	0	2.301	**1.301**	**−1.000**	Y	Redetachment
7	10	8	Y	N	0	2.301	**1.000**	**−1.301**	N	
8	6	7	Y	N	0	1.854	**1.301**	**−0.553**	N	
9	17	5	Y	N	0	0.155	**0.155**	**0.000**	N	
10	8	7	Y	N	0	0.824	**0.097**	**−0.727**	Y	Nicked retinal blood vessel
11	4	4	Y	N	1	0.699	**0.699**	**0.000**	N	
12	7	4	Y	N	2	0.301	**0.301**	**0.000**	N	
13	19	4	Y	N	1	1.854	**1.301**	**−0.553**	N	
14	10	5	Y	N	1	1.854	**1.301**	**−0.553**	N	
15	4	3	Y	N	0	1.854	**0.155**	**−1.699**	N	
16	16	6	Y	N	0	0.000	**0.000**	**0.000**	N	
17	18	7	Y	N	0	2.602	**0.699**	**−1.903**	N	
18	12	4	y	N	0	1.301	**0.301**	**−1.000**	N	
19	0	6	Y	N	0	0.301	**0.301**	**0.000**	Y	Hypotony
20	20	6	Y	N	1	1.854	**1.000**	**−0.854**	N	
21	4	2	Y	N	1	1.854	**0.699**	**−1.155**	N	
22	8	3	Y	N	0	0.301	**0.000**	**−0.301**	N	
23	14	2	Y	N	0	2.602	**1.000**	**−1.602**	N	
24	9	7	Y	N	0	0.301	**0.155**	**−0.146**	N	
25	4	4	Y	N	0	0.174	**0.097**	**−0.077**	N	
26	8	4	Y	N	0	0.174	**0.000**	**−0.174**	N	
27	4	3	Y	N	0	1.301	**0.699**	**−0.602**	N	
28	4	5	Y	N	0	2.301	**1.301**	**−1.000**	Y	IOP elevation
29	27	5	Y	N	0	0.699	**0.824**	**0.125**	N	
30	38	2	Y	N	0	0.699	**0.398**	**−0.301**	Y	IOP elevation
31	5	3	Y	N	1	2.301	**2.301**	**0.000**	N	
32	4	4	Y	N	0	2.301	**2.301**	**0.000**	N	
33	0	7	Y	N	0	0.699	**0.699**	**0.000**	Y	Hypotony
34	14	3	Y	N	0	1.301	**0.523**	**−0.778**	N	
35	18	5	Y	N	0	0.174	**0.000**	**−0.174**	N	
36	2	8	Y	N	1	2.602	**2.602**	**0.000**	Y	Hypotony
37	15	8	Y	N	1	1.854	**1.000**	**−0.854**	N	
38	12	1	Y	N	0	0.398	**0.260**	**−0.138**	N	
39	16	3	Y	N	0	0.456	**0.222**	**−0.234**	N	
40	8	6	Y	N	0	0.398	**0.301**	**−0.097**	N	
41	8	4	Y	N	0	0.481	**0.097**	**−0.385**	N	
42	13	4	Y	N	0	1.000	**1.000**	**0.000**	N	
43	34	5	Y	N	0	1.000	**1.000**	**0.000**	Y	IOP elevation
44	23	3	Y	N	0	0.301	**0.155**	**−0.146**	N	
45	10	3	Y	N	0	0.301	**0.301**	**0.000**	N	
46	16	2	Y	N	0	0.155	**0.301**	**0.146**	N	
47	8	5	Y	N	0	1.854	**1.301**	**−0.553**	N	
48	15	7	Y	N	0	0.000	**0.000**	**0.000**	N	
49	11	2	Y	N	0	1.301	**0.481**	**−0.820**	N	
50	30	6	Y	N	0	1.301	**0.301**	**−1.000**	Y	IOP elevation
**Mean**	***12.0***	***4.5***				***1.237***	***0.713***	***−0.524***		
**SD**	***8.3***	***1.9***				***0.881***	***0.656***	***0.562***		

*Abbreviations*: *AE* Adverse Events, *AM* Amniotic membrane, *DCVA* Distance corrected visual acuity, *DIS* Dislocated Intraocular lens, *ERM* Epiretinal membrane, *FAX* Fluid air exchange, *GT* Gas tamponade, *ILM* Internal limiting membrane, *LX* Endolaser photocoagulation, *INTRAOP* Intraoperative, *MD* Membrane dissection, *MH* Macular hole, *OR* Operative, *PPV* Pars plana vitrectomy, *PREOP* Preoperative, *RIOL* Removal of dislocated IOL, *RLF* Retained lens fragments, *RRD* Rhegmatogenous retinal detachment, *SIOL* Secondary intraocular lens implantation, *ST* Silicone oil tamponade, *TRD* Traction retinal detachment, *VH* Vitreous hemorrhage, *VIT* Vitritis

The surgical objectives were attained in all eyes. At the 3-month postoperative visit, the mean CDVA improved (*P* < 0.05) from 1.24 to 0.71 logMAR units. CDVA improved by 2 lines or more in 56%, remained unchanged in 40%, and decreased by 2 lines or more in 4% of eyes.

The mean TOT was 1891 ± 890 (range, 510–3930) seconds. The mean CVT, SVT, and TVT were 204 ± 120 (range, 60–594), 330 ± 320 (range, 90–1782) and 534 ± 389 (range, 155–2376) seconds, respectively. The mean number of surgical steps was 4.3 ± 1.5 (range, 1–9); the mean number of times ancillary instruments were placed in each eye was 4.5 ± 1.9 (range, 1–8) times.

Intraoperative AE included an iatrogenic retinal break in one eye (2%) and nicked retinal vessel in another eye (2%) which was easily controlled by increasing IOP. Postoperative AE included IOP elevation in 4 eyes (8%), hypotony in 3 eyes (6%) and recurrent RRD, in one eye with long axial length (2%). None of the eyes required sclerotomy suturing.

PPV using the 25G BTCP was well-tolerated. The mean postoperative Day 1 pain grading was 0.2 ± 0.5 (range, 0–2). Forty-one patients (82%) reported no pain, 2 (4%) reported mild pain, and 1 patient (2%) reported moderate pain.

The number of surgical steps demonstrated a positive correlation with TOT (*p* < 0.05), number of ancillary instruments used (*p* < 0.05), and postoperative Day 1 IOP (*p* < 0.05). The number of times ancillary instrumentation was used demonstrated a positive correlation with TOT (*p* < 0.05). Postoperative day 1 IOP was not correlated to TOT, number of ancillary instruments used, nor to CVT or SVT. Postoperative pain scores and CDVA change after 3 months were unrelated to other variables (Table 2).

**Table 2:** Correlation Analysis of Surgical Variables

**A. Number of Surgical Steps Versus**	**r value**	***p*value**
***Total Operative Time***	***0.593***	< 0.0001*
***Number of Times Ancillary Instrument Used***	***0.483***	0.0004*
***Postoperative Day 1 IOP***	***0.293***	0.0388*
Shave Vitrectomy Time	0.195	0.1757
Core+Shave Victrectomy Time	0.195	0.1757
Core Vitrectomy Time	0.116	0.4241
logMAR change	0.046	0.7532
**B. Number of Times Ancillary Instrument Used Versus**	**Pearson r**	***p*value**
***Total Operative Time***	***0.535***	0.0001*
Postoperative Day 1 IOP	−0.066	0.6476
Core Vitrectomy Time	0.044	0.7599
logMAR change	0.018	0.9006
Shave VItrectomy Time	−0.030	0.8362
Core+Shave Victrectomy Time	−0.011	0.9397
**C. Postoperative Day 1 IOP Versus**	***r*value**	***p*value**
***Number of Surgical Steps***	***0.293***	0.0388*
Total Operative Time	0.178	0.2158
Number of Times Ancillary I trument Used	−0.066	0.6476
Shave Time	0.050	0.7325
Core+Shave Victrectomy Time	0.047	0.7469
Core Time	0.020	0.893
**D. Postoperative Pain Score Versus**	***r*value**	***p*value**
Core Vitrectomy Time	0.156	0.2794
Number of Surgical Steps	−0.153	0.2897
Postoperative Day 1 IOP	−0.137	0.3432
Number of Times Ancillary I trument Used	0.093	0.5191
Core+Shave Victrectomy Time	0.0867	0.5492
Total Operative Time	−0.023	0.8734
Shave Vitrectomy Time	−0.002	0.9914
logMAR change	−0.066	0.6497
**E.Total Vitrectomy Time (Core + Shave) Versus**	**r value**	***p*value**
***Total Operative Time***	**0.5608**	0.0001*
logMAR change	0.252	0.0773
Number of Surgical Steps	0.195	0.1757
Postoperative Day 1 IOP	0.047	0.7469
Number of Times Ancillary I trument Used	−0.011	0.9397
Postoperative Pain Score	−0.023	0.8734
**F. logMAR change**	***r*value**	***p*value**
Total OR TIME	0.234	0.1019
Shave Time	0.224	0.1174
Core Time	0.223	0.1194
PPV	0.252	0.0773
Number of Steps	0.046	0.7532
Number of instruments	0.018	0.9006
Pain	−0.066	0.6497

* significant

*Abbreviation*: *IOP* Intraocular pressure

## Discussion

MIVS, or transconjunctival sutureless vitrectomy surgery as first described by Fujii and colleagues, has become the standard of care for VR surgery [6, 7]. Incremental technological improvements such as higher cutting speeds, better fluidics, and cutter probe modifications such as the beveled-tip design used in this study, continue to enhance the effectiveness and safety of PPV. This open-label, prospective case series demonstrated that a high speed, 25G, BTCP as utilized by multiple surgeons effectively and safely achieved the surgical objectives for common VR conditions. Additionally, this study uniquely explored the relationship of surgical efficiency parameters such as operative time, usage of ancillary instrumentation and their relationship to surgical complexity.

Using this system, we observed significant visual acuity improvement of 0.53 ± 0.56 logMAR units (18.36 ± 19.61 ETDRS letters, *P* < 0.001) 3 months after surgery. The magnitude of improvement in postoperative visual outcomes observed here is comparable to results of studies using similar gauge instrumentation [8–13]. Mitsui et al. prospectively compared 27G and 25G vitrectomy systems for eyes with ERM and measured visual acuity across 3 follow-up visits and found statistically significant improvements in the 2 groups at each postoperative visit [8]. An approximately 10 letter gain was noted at 3 months among those undergoing 25G vitrectomy. A retrospective study of ERM surgeries by Naruse et al. also reported visual gains of 4.6 ± 13.4 letters in the third postoperative month when using 25G systems [9]. In 5 patients with isolated ERM, the average visual improvement in our study was approximately 4 ± 4.18 letters. Two studies compared the flat tip, 7500 cm 25G and 27G systems in cases of RRD [10, 11]. Rizzo et al. reported an improvement of 30 letters 3 months after surgery while Sborgia et al. similarly reported an improvement of 35 letters. These were similar to our subset of 9 patients with a sole diagnosis of RRD who had an improvement of 19.22 ± 15.10 letters by the third postoperative month. A study by Naruse et al. reported an improvement of 17.5 ± 28.1 letters among patients operated on for proliferative diabetic retinopathy using 25G flat tip 5000 cpm system [12]. In our subset of patients with vitreous hemorrhage and tractional retinal detachment, the improvement in vision in 3 months was 26.5 ± 15.7 ETDRS letters (Table 3).

**Table 3:** Comparison of 3-month postoperative visual acuities across different 25G studies

Indication	Instrumentation	Visual Acuity Gain (ETDRS Letters)
RRD	25G, beveled tip, 10,000 cpm (Current Study, *n* = 9)	19.22 ± 15.10
	25G, flat tip, 7500 cpm (Sborgia et al., 2019) [11]	35
	25G, flat tip, 7500 cpm (Rizzo et al., 2017) [10]	30
ERM	25G, beveled tip, 10,000 cpm (Current Study, *n* = 5)	4 ± 4.18
	25G, flat tip, 5000 cpm (Naruse et al., 2017) [9]	4.6 ± 13.4
	25G, flat tip, 5000 cpm (Mitsui et al., 2016) [8]	10
PDR	25G, beveled tip, 10,000 cpm (Current Study, *n* = 4)	26.50 ± 15.70
	25G, flat tip, 5000 cpm (Naruse et al., 2019) [12]	17.5 ± 28.1

*Legend*: *RRD* Rhegmatogenous retinal detachment, *ERM* Epiretinal membrane, *PDR* Proliferative diabetic retinpoathy

The results of this study suggest that employment of the 25G BTCP may decrease operative times. We observed that mean and total operative times for individual phases of the PPV procedure were closer to the lower end of the ranges reported by similar studies using 25G probes (Table 4). Total operative time was observed to be correlated with the number of surgical steps and ancillary instruments used. These 3 closely-related variables indicating surgical complexity were uniquely quantified in this study. As longer operative durations and frequent instrument entry and exit may increase the risk for complications, new advances that shorten operating and recovery times, enhance surgeon productivity, and lower procedural costs are always welcome. We understand, however, that because case complexity and surgeon skill can independently influence operative time, a direct comparison of surgical efficiency across different practices and time periods is difficult and should be done with caution.

**Table 4:** Comparison of total operative and vitrectomy times across different 25G studies

Surgical Parameter	Indication	Instrumentation	Time (Minutes)
Total Operative Time	RRD	25G, beveled tip, 10,000 cpm (Current Study)	39.0 ± 14.2
		25G, flat tip, 7500 cpm (Sborgia et al., 2019) [11]	64.4 ± 9.5
	ERM	25G, beveled tip, 10,000 cpm (Current Study)	22.4 ± 4.1
		25G, flat tip, 5000 cpm (Naruse et al., 2017) [9]	32.7 ± 10.1
		25G, flat tip, 5000 cpm (Mitsui et al., 2016) [8]	16.1 ± 9.3
Total Vitrectomy Time	RRD	25G, beveled tip, 10,000 cpm (Current Study)	14.4 ± 5
		25G, flat tip, 7500 cpm (Sborgia et al., 2019) [11]	20.8 ± 3.8
		25G, flat tip, 7500 cpm (Rizzo et al., 2017) [10]	19.6 ± 7.3
	ERM	25G, beveled tip, 10,000 cpm (Current Study)	4.7 ± 1.7
		25G, flat tip, 5000 cpm (Mitsui et al., 2016) [8]	6.2 ± 2.7
	Various Indications	25G, beveled tip, 1000 cpm (Current Study)	8.9 ± 6.5
		25G, flat tip, 7500 cpm (Rizzo et al., 2011) [14]	18.4 ± 9.6
		25G, flat tip, 5000 cpm (Rizzo et al., 2011) [14]	26.4 ± 14.6

*Legend*: *RRD* Rhegmatogenous retinal detachment, *ERM* Epiretinal membrane

**Fig. 2:**
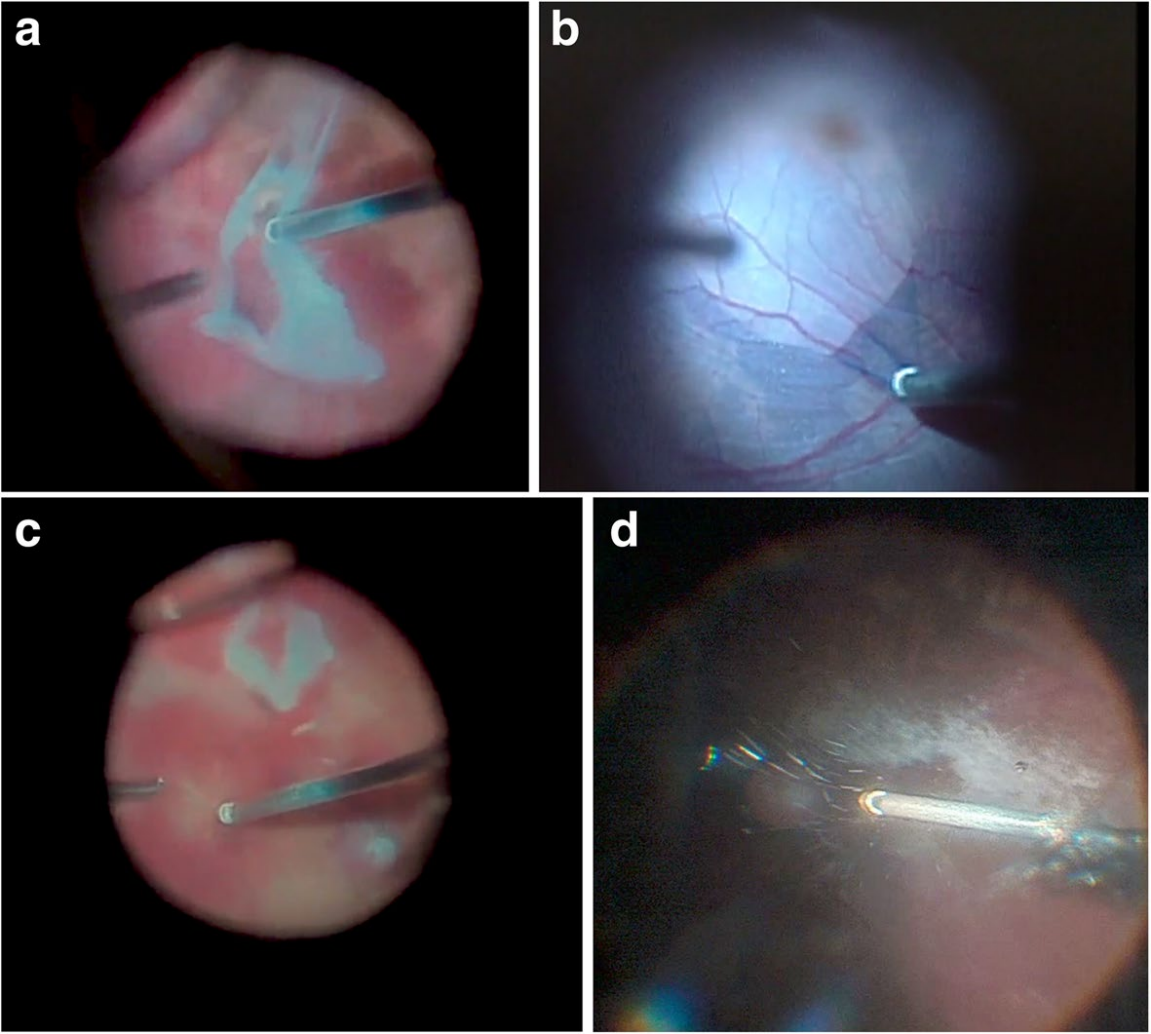
Surgical microscope view of 25-gauge, beveled-tip probe utilized to engage pre-retinal fibroproliferative tissues during membrane dissection (**A**), to peel the internal limiting membrane and overlying epiretinal membrane after staining with a combination of Brilliant Blue and trypan blue dye (**B**), and to remove a thin layer of heme near the retinal surface using aspiration mode only (**C**). An even, laminar flow of triamcinolone acetonide-stained vitreous into the port-opening can be achieved using open-biased duty cycle (**D**)

The BTCP features a port opening that is significantly closer to the distal tip (0.009 in.), half the distance of conventional flat-tip probes (0.018 in.). The multifunctional capabilities of this unique probe geometry have been supported by laboratory and clinical studies [15, 16]. This shortened port-tip distance improves access to surgical tissue planes and facilitates aspiration of preretinal and subretinal materials and has the potential to improve surgical efficiency. With this working distance, an improved ability of the dual-pneumatic probe to control fluidics, and enhanced surgeon control via machine software and hardware, the BTCP can be used in a multifunctional role to perform many steps currently being done using ancillary instruments. For example, the BTCP tip can be used to grasp and manipulate pre-retinal and even thin internal limiting membranes thus minimizing the use of tissue forceps. The fine tip can be insinuated into preretinal membranes and be used as a scissors to nibble and cut fibrovascular tissues. We have also used the BTCP as a flute needle to aspirate fluid during complete fluid-air exchange. The BTCP tip can also be brought closer to the retinal surface to aspirate thick, coagulated heme and to minimize the need for refluxing fluid to blow off pre-retinal material. Fig. 2 shows how various surgical steps can be achieved by using the cutter probe alone (See Video 1 Supplemental Digital Content, Surgical Maneuvers). Several maneuvers such as the “lift-and-shave” and “shovel-and-cut” techniques have been described to enable surgeons to dissect diabetic membranes with greater facility which may also lessen the use of ancillary instruments [16, 17]. The smaller 27G BTCP may further improve tissue access but may also decrease vitreous flow.

Although we were able to perform several maneuvers using the ultra-high speed BTCP in place of forceps, scissors and soft-tip cannulas, it should be emphasized that a beveled-tip cutter is not optimal for these steps and should not be used whenever more proper instrumentation is available. Ancillary instruments are preferable for many surgical steps, such as retinal scissors for dissecting adherent or broad-based diabetic membranes and membranes on detached and atrophic retina, soft tip cannulas for FAX for a less traumatic aspiration over the macula and optic nerve head, and retinal forceps for initiating pinch-and-peel ERM and ILM peeling. The BTCP can however be used complementarily with a second instrument for manipulating and dissecting tissues, such as combining with a retinal pick to lift adherent posterior vitreous under chandelier lighting, or with retinal scissors for dissection of dense membranes. We find that a learning curve exists for utilizing BTCP in a multifunctional role.

We observed no additional safety concerns using the BTCP. Common intraoperative complications such as retinal and vitreous hemorrhage, iatrogenic breaks, and postoperative pressure changes occurred at a similar frequency as in previous reports [18–20]. These were readily addressed using conventional measures such as laser photocoagulation, IOP elevation, gas tamponade and postoperative medications. The incidence of immediate postoperative hypotony and IOP elevation were also similar to those reported in previous literature using conventional MIVS [8–12, 19, 20]. The use of a smaller 27G probe may mitigate the risks for postoperative hypotony in complex cases [21].

A recent meta-analysis has reported redetachment rates of about 20.9% after primary PPV [22]. Smaller gauge instrumentation with improved vitreous cutting and fluidics may minimize iatrogenic tears and postoperative RD by reducing pulsatile traction, wound leaks, vitreous-wound incarceration, iatrogenic retinal trauma, and facilitating pre-retinal traction membrane removal. Re-detachment occurred in 1 eye treated for RRD which was at a higher risk because of very long axial length. This was successfully treated with repeat PPV and silicone oil tamponade. No cases of endophthalmitis were observed in this series. The procedure was well tolerated by majority of patients who reported absence of pain within the first 24 h after surgery.

The results of this study lend evidence to the ability of ultra-high speed, 10,000 cpm probes to shorten vitreous gel removal time. In vitro studies have reported a proportional relationship between vitreous flow and cutting speeds when using 50/50 or biased closed duty cycle across different cutter probe gauges [23, 24]. The amount of aspirated vitreous collagen material is related to cutter characteristics as summarized in the equation:

Theoretical Vitreous Chunk Length = Flow Rate through Aspiration Line / (Cutter Port Surface x Cut Rate) [25].

By utilizing ultra-high cut rates, vitreous can be quickly segmented into smaller pieces facilitating smoother, less turbulent aspiration even when using smaller diameter lumen [24]. Higher cut rates can also enhance surgical precision and safety by minimizing pulsatile vitreous movement and avoiding iatrogenic retinal breaks.

Flow dynamic studies in porcine eyes have demonstrated faster aspiration and reflux velocities when using BTCP [15]. Beveled-tip geometry has been reported to prolong high aspirating pressures during the duty cycle and lower tip turbulence at the port opening [22]. The improved flow dynamics of the BTCP contribute to faster vitreous aspiration which may account for the shorter vitrectomy times observed in this study.

Our prospective study design included standardized measurement of efficacy and efficiency variables, such as operative times, number of surgical steps and ancillary instrument use. We also conducted correlation analysis to identify associations among variables and established a direct relationship between total operative time and number of ancillary instruments used and number of times such instruments were used. This indicates that for more complex cases, surgeons employ adjunctive instrumentation more frequently. Further research is therefore needed to explore whether new technologies, such as BTCP or other cutter probe configurations, that perform multiple functions may help decrease operative times and complications.

This study demonstrates the effectiveness of a BTCP for the surgical treatment of common VR indications in producing generally similar outcomes as those achieved using earlier cutter probe configurations. Furthermore, while acknowledging the limitations of comparing studies from different institutions and population groups, our results suggest that operative times may be reduced using a 25-gauge, 10,000 cpm, BTCP system. The TOT and TVT observed in this study, for a given indication, were shorter compared to the majority of studies wherein these temporal parameters have been reported (Table 4). Novel surgical parameters quantified in this study included the number of ancillary instruments used as well as the number of instrument exchanges performed. We believe similar structural and functional outcomes were achieved using shorter operative durations because of unique features of the BTCP. The shorter surgical durations may allow a practice to perform more procedures during a given day and increase efficiency. Reduction in ancillary instrumentation use may also result in cost savings for the practice or reduce the risk of infection brought about by frequent instrument exchanges and imperfect sterilization of reusable instrumentation.

It should be recognized that while the BTCP may substitute for other instrumentation, in many instances, traditional ancillary instrumentation is still optimal for a specific task and are preferentially used such as with initiation of and extensive membrane peeling, bimanual surgery, drainage of subretinal fluid, pre- and subretinal fluid injections, removal of subretinal removal of very large nuclear fragments or foreign objects.

Our study has several limitations which should be considered in analyzing our results. The study was descriptive in that we used only a single type of MIVS cutter probe without a control group which does not allow us to directly compare surgical results with other available probes, machines, and settings. Given the small patient population, our study is not sufficiently powered to detect rare events such as endophthalmitis, choroidal bleed, and subretinal migration of tamponade agents. The surgeries were done by 3 different vitreoretinal specialists to account for differences in surgical preferences and techniques, although we understand that our single center design may not be representative of the greater surgical community. A larger surgeon population should also be able to decrease potential data collection bias. Larger, randomized, controlled trials involving multiple surgeons are needed to compare the different probe designs. We recognize that operative duration and clinical outcomes are influenced not only by the type of MIVS utilized but also by other factors such as stage of the disease, operative complexity, patient cooperation, quality of surgical assistants and other operating room equipment such as surgical microscope and viewing systems. The results of this investigation provide basis for larger studies to fully examine the value of BTCP across a broad range of conditions. In conclusion, an ultra-high speed, 25G, BTCP appears effective and safe for treating a variety of VR conditions and has the potential to reduce the use of ancillary instrumentation and operative time in pars plana vitrectomy. Further studies are needed to fully elucidate the advantages and limitations of this novel probe design.

## Supplementary Information

**Additional file 1.** Supplemental Digital Content. Video demonstrating 25-gauge, beveled tip cutter probe during diabetic retinopathy surgery.

## Data Availability

All data generated or analysed during this study are included in this published article.
